# Adherence to Treatment and Level of Satisfaction Among Saudi Hypertensive Patients: A Multi-City Study

**DOI:** 10.7759/cureus.20189

**Published:** 2021-12-05

**Authors:** Eid H Alshahrani, Reham S Aljohani, Angham A Sahli, Wejdan S Alruwaili, Ibrahim A Almohini, Hind Almodaimegh

**Affiliations:** 1 Otolaryngology - Head and Neck Surgery, King Abdullah Hospital, Ministry of Health, Bisha, SAU; 2 Pediatrics, King Abdullah Specialized Children Hospital, National Guard Health Affairs, Riyadh, SAU; 3 Internal Medicine, King Abdulaziz Medical City, National Guard Health Affairs, Riyadh, SAU; 4 Ophthalmology, King Khaled Hospital, Ministry of Health, Hail, SAU; 5 General Surgery, Prince Salman bin Mohammed Hospital, Ministry of Health, Al-Dilam, SAU; 6 Pharmacology and Therapeutics, College of Pharmacy, King Abdullah International Medical Research Center/King Saud bin Abdulaziz University for Health Sciences, Riyadh, SAU

**Keywords:** adherence, blood pressure control, high blood pressure, patients' satisfaction, hypertension, saudi arabia

## Abstract

Background and objective

Hypertension (HTN) is a common disease among patients who visit primary healthcare clinics. Uncontrolled HTN is associated with increased morbidity and mortality; therefore, awareness of its risk factors and adherence to treatment can lead to better control of HTN. In this study, we aimed to determine the level of adherence to treatment and satisfaction among Saudi hypertensive patients.

Methods

In this cross-sectional study, we used an interviewer-administered questionnaire among hypertensive patients aged 40 years or older who attended primary healthcare centers in the five main regions within Saudi Arabia. We excluded patients with mental disorders, dementia, or those unable to provide consent to participate. The estimated sample size was 384 patients as calculated by Raosoft® based on the AlNozha study (prevalence of HTN in Saudi Arabia is 26.1%). The questionnaire included questions about demographic data, awareness about HTN risk factors and complications, adherence to treatment, and patient satisfaction along with the information related to management from their physicians. Microsoft Excel was used for data entry, and SPSS Statistics version 23 (IBM, Armonk, NY) was used for statistical analysis.

Results

Of the 384 hypertensive patients recruited, most were in the age group of 40-49 years (36.5%), and the majority of them were female (75%). Most patients (n=305, 79.4%) reported good adherence to the management plan. Also, most of the patients (73.4%) were satisfied in terms of receiving simplified information and justification of the management plan from their physicians.

Conclusions

A good level of satisfaction was observed among patients of HTN in Saudi Arabia with respect to information, simplification, and justification of treatment plans provided by health professionals. This high satisfaction level accounts for the high adherence to the treatment plan.

## Introduction

Hypertension (HTN) is a chronic disease characterized by an elevation of arterial blood pressure, which negatively affects the physiological state of the body [1]. According to the World Health Organization (WHO), the prevalence of HTN rose to affect approximately 1.13 billion in 2015 [2]. Moreover, a study by Tailakh et al. has revealed that the direct consequences of HTN accounted for almost four million deaths per year [3]. HTN is highly associated with the increasing age of the population [4]. Most HTN patients are asymptomatic and are diagnosed incidentally during routine clinic visits [2].

Unfortunately, some patients are diagnosed with HTN very late in the course of the ailment, when the complications are already present. These complications can negatively affect the quality of life and may even be life-threatening [5]. According to the Eighth Joint National Committee (JNC 8) guidelines, the target blood pressure for the general population is <140/90 mmHg for patients aged below 60 years and <150/90 for those above 60 years in age [6]. Many studies have shown that a low educational level, obesity, physical inactivity, and high salt intake represent significant risk factors for HTN [7-9].

In Saudi Arabia, the Ministry of Health (MOH) has estimated the incidence of HTN among Saudis to be 15.1% [10]. The Global Burden of Disease 2017 (GBD 2017) study illustrated that HTN was a high risk factor for mortality among the Saudi population [11]. From 2010 to 2017, HTN persisted as one of the most serious health problems in Saudi Arabia [11]. Some studies have reported a gender-wise difference with respect to the control of HTN; women reportedly have higher control than men, especially since they visit healthcare providers more often for birth control and gynecological surveillance, which increases their exposure to screening and educational opportunities, while men seek healthcare or visit health facilities at a significantly lower rate in general [12-14].

There have been significant attempts to control HTN and increase awareness about it among the general population, especially among those at high risk of developing disease complications, which are associated with uncontrolled levels of blood pressure [15]. Many strategies have been implemented to achieve target blood control. One study reveals that with lifestyle modifications and reduced salt intake, 84% of the participants were able to attain their target blood pressure levels [9]. Another study has suggested that weight reduction was a significant factor in achieving blood pressure control [16]. Our study aimed to measure the adherence to treatment among Saudi hypertensive patients and their level of satisfaction with the health information and medical management they receive from the physicians.

## Materials and methods

Study setting, study design, and sampling technique

A cross-sectional study based on an interviewer-administered questionnaire was conducted among hypertensive patients who attended primary healthcare centers in five main regions within Saudi Arabia (central, eastern, northern, western, and southern). We included patients who were 40 years or older and diagnosed with essential HTN. We excluded all patients who were concurrently diagnosed with mental disorders or dementia. The estimated sample size was 384 patients as calculated by Raosoft® based on AlNozha's study (prevalence of HTN in Saudi Arabia is 26.1%) with a confidence level of 95%, a confidence interval of 5%, and a response distribution of 50% [17]. The sampling technique employed was convenient sampling. The study was approved by the Institutional Review Board (IRB) Office, King Abdullah International Medical Research Center (KAIMRC), Riyadh, Saudi Arabia, with the approval memo ref. no. IRBC/1270/18.

Data collection instruments, measurements

To validate the questionnaire, multiple steps were performed. Firstly, to ensure that the content of the questionnaire correctly matched the study objectives and handled the topic well, the questionnaire was created by an internal medicine consultant experienced in the field of research. Then, the questionnaire was reviewed by three internal medicine consultants, and they approved it after modifying it and adjusting it for enhancement. The questionnaire was created first in English, then it was translated by an expert linguist to Arabic, which was then translated back to English to ensure high accuracy and correct use of words and grammar. Lastly, a pilot study was launched involving 25 participants where the questionnaire was examined in an interview to make sure the participants had a correct and proper understanding of the questionnaire. Cronbach's alpha was used to validate the Likert scale used in the questionnaire and yielded a result of (0.81), which confirmed its statistical validity.

Data management and analysis

We used Microsoft Excel for data entry and SPSS Statistics version 23 (IBM, Armonk, NY) for statistical analysis by which descriptive statistical evaluation (frequency, percentages, mean, and standard deviation) were performed. We utilized Cronbach's alpha to measure the internal consistency, which was determined to be 0.75. Chi-square test and independent t-test were also performed to test for the presence of significant relationships.

## Results

The total number of participants was 384, 75% of whom were female. Most patients were in the age group of 40-49 years (36.5%). Regarding the geographical origins of the patients, most of them hailed from the southern region (n=122, 31.8%). Most of the patients were illiterate (n=116, 30.2%). We found diabetes among 123 (32%) patients in the cohort (Table 1).

**Table 1:** Demographic variables

Variables	Categories	N (%)
Geographical region	Central	69 (17.9)
	Eastern	81 (21.1)
	Northern	81 (21.1)
	Western	31 (8.1)
	Southern	122 (31.8)
Age group	40-49 years	140 (36.5)
	50-59 years	128 (33.3)
	60-69 years	80 (20.8)
	≥70 years	36 (9.4)
Gender	Male	96 (25.0)
	Female	288 (75.0)
Educational level	Illiterate	116 (30.2)
	Primary school	58 (15.1)
	Elementary school	51 (13.3)
	Secondary school	75 (19.5)
	University	78 (20.3)
	Higher education	6 (1.6)
Comorbidities	Chronic kidney disease	11 (2.9)
	Diabetes mellitus	123 (32.0)
	Chronic heart disease	21 (5.5)
	Multiple comorbidities	30 (7.8)

Regarding adherence to the treatment plan, more than two-thirds of the participants (n=305, 79.4%) stated that they adhered to their management plan, and among them, female patients formed the majority (n=227, 74.4%). Seventy-eight (81.25%) males adhered to the treatment plan, while 227 (78.8%) females adhered. Regarding the relationship between gender and adherence to treatment, no significant difference was found between males and females in neither the rate of complying with the treatment plan nor in the extent of adherence within the groups who reported adhering to the treatment (Table 2).

**Table 2:** Relationship between gender and adherence to treatment

Factor	Gender		P-value
	Female	Male	
Follow treatment plan			0.61
Yes, n (%)	227 (78.8%)	78 (81.2%)	
No, n (%)	61 (21.2%)	18 (18.8%)	
The extent of adherence to the treatment plan			0.981
Mean	72.43	72.5	
Standard deviation	25.04	24.5	

In terms of the factors associated with satisfaction with the consultation, a significant association was found between age and satisfaction (p=0.003), where it was observed that the older the patients the higher the rate of satisfaction toward the consultation. A significant association was also found between education and satisfaction (p=0.005), where it was seen that the higher the education, the lower the rate of satisfaction. A significant association was also found between comorbidities and satisfaction (p<0.001), where the highest rate of satisfaction was found in those with multiple comorbidities, and the lowest rate of satisfaction was seen in patients with chronic kidney disease (Table 3).

**Table 3:** Factors associated with satisfaction with the consultation *Significant at the level of 0.05

Factor	Satisfaction level			P-value
	Dissatisfied, n (%)	Neutral, n (%)	Satisfied, n (%)	
Age group				0.003*
40-49 years	7 (5%)	42 (30%)	91 (65%)	
50-59 years	4 (3.1%)	33 (25.8%)	91 (71.1%)	
60-69 years	4 (5%)	9 (11.3%)	67 (83.8%)	
≥70 years	2 (5.6%)	1 (2.8%)	33 (91.7%)	
Gender				0.318
Female	12 (4.2%)	69 (24%)	207 (71.9%)	
Male	5 (5.2%)	16 (16.7%)	75 (78.1%)	
Education				0.005*
Illiterate	4 (3.4%)	14 (12.1%)	98 (84.5%)	
Primary school	2 (3.4%)	10 (17.2%)	46 (79.3%)	
Elementary school	1 (2%)	11 (21.6%)	39 (76.5%)	
Secondary school	5 (6.7%)	18 (24%)	52 (69.3%)	
University	5 (6.4%)	29 (37.2%)	44 (56.4%)	
Higher education	0 (0%)	3 (50%)	3 (50%)	
Comorbidities				<0.001*
Chronic kidney disease	3 (27.3%)	2 (18.2%)	6 (54.5%)	
Diabetes	2 (1.6%)	27 (22%)	94 (76.4%)	
Chronic heart disease	0 (0%)	3 (14.3%)	18 (85.7%)	
Multiple comorbidities	1 (3.3%)	3 (10%)	26 (86.7%)	

**Figure 1:**
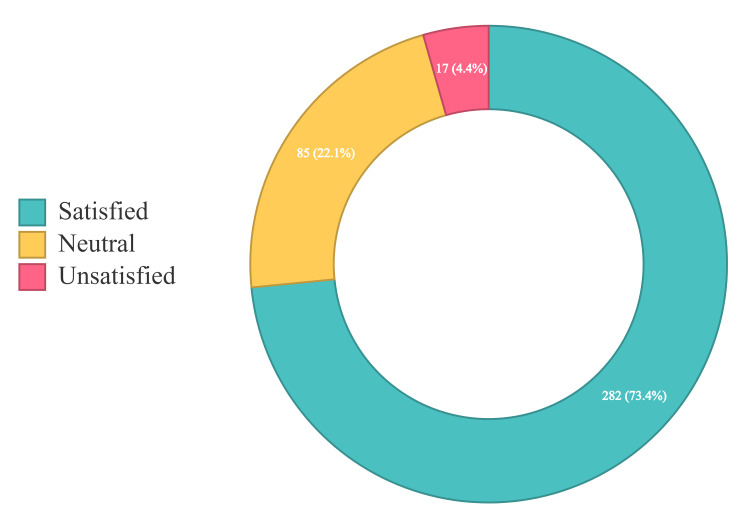
Satisfaction level of patients with the information on the treatment plan given by their physicians

Approximately three-quarters of the patients (73.4%) were satisfied with the information, its simplification, and justification regarding their treatment plan as provided by the physician. The rest of the patients were either unsatisfied (4.4%) or neutral (22.1%) (Figure 1).

A significant number of patients did not believe that smoking, physical inactivity, and obesity are major HTN risk factors, while only 20%, 32.5%, and 40.8% of patients believed that smoking, physical inactivity, and obesity are risk factors for HTN, respectively. Regarding the patients' perception of the common complications of HTN, we found that 46.4% and 42.7% of patients believed that stroke and cardiovascular diseases were the main complications, respectively. A small proportion believed that vision problems (29.9%) and kidney diseases (27.9%) were the main complications (Table 4).

**Table 4:** Perception of hypertensive patients about their disease risk factors and complications

Variables	Categories	Answer: n (%)
Do you know if these are risk factors for HTN or not?	Smoking	Yes: 77 (20.1)
		No: 307 (79.9)
	Obesity	Yes: 157 (40.9)
		No: 227 (59.1)
	Physical inactivity	Yes: 125 (32.6)
		No: 259 (67.4)
	Stress	Yes: 276 (71.9)
		No: 108 (28.1)
	High salt intake	Yes: 186 (48.4)
		No: 198 (51.6)
Do you know if these are complications for HTN or not?	Kidney diseases	Yes: 107 (27.9)
		No: 277 (72.1)
	Stroke	Yes: 178 (46.4)
		No: 206 (53.6)
	Vision problems	Yes: 115 (29.9)
		No: 269 (70.1)
	Cardiovascular diseases	Yes: 164 (42.7)
		No: 220 (57.3)

## Discussion

HTN is a serious non-communicable medical condition in many parts of the world. Efforts to control HTN and alleviate the related health risks can be undermined by the insufficient level of knowledge and awareness of these risks among the people. Our study included 384 participants. Among them, the majority were in the age group of 40-49 years (36.5%), and 75% of the respondents were females. Our findings also showed that 30.2% of the respondents were illiterate. Adherence to the treatment plan was higher among males (81.25%) when compared to females (78.8%). Regarding the level of satisfaction with the simplification of information received from health practitioners, the majority of the participants (73.4%) were satisfied. Our findings also showed that 5.5% of the respondents had chronic heart diseases, 7.8% had multiple comorbidities, and 2.9% had renal diseases.

The hypertensive profile of the patients in the present study is consistent with other studies in the literature regarding the connection between HTN and diabetes. The current study revealed that diabetes was reported in 32% of hypertensive patients. The association between diabetes and obesity has been reported in other studies as well. For example, according to a study conducted in Saudi Arabia in 2018, the coexistence of diabetes and HTN was reported in 56.4% of the cases [18].

Our findings indicated good adherence to the treatment plan, as 79.4% of the respondents reported complying with it, and 81.2% of those who adhered to the treatment plan were males. These findings are consistent with a study from Ajman, UAE in 2016, where adherence to treatment was reported among 84.4% of the patients [19]. However, in a study conducted in the US, the level of adherence to antihypertensive drugs (68.8%) was slightly lower than that in this study [20]. The discrepancy between our findings and the findings of the US study can be explained by the difference in the nature of the sample population: their study sampled the general public, whereas our sample only included patients attending primary healthcare centers. Patients’ awareness of the importance of adherence to treatment is essential to achieve good control of blood pressure [21]. Most of the participants in this study (73.4%) were satisfied with the information and explanation of the treatment plan provided by the health workers. This result may explain the high level of adherence to the treatment plan reported in this study. This finding is consistent with a study conducted in Ann Arbor, MI, which established a link between poor adherence to treatment and a lack of information from health professionals [22].

Regarding the HTN risk factors, stress, obesity, and lack of physical activity were the most quoted risk factors in this study, at 71.9%, 40.9%, and 32.6%, respectively [23]. This result is consistent with two studies from the US, which reported that stress, obesity, and diabetes were the major risk factors for HTN [23,24]. Public awareness of HTN and its risk factors is necessary to facilitate the job of health professionals to prevent and treat high blood pressure [21,23].

This study has yielded significant results regarding the satisfaction of the hypertensive patients with their physicians and adherence to the treatment administered. However, the study has a major limitation: the possibility of recall bias. Recall bias might have crept in due to the nature of the survey, which involved asking the patients to answer questions about their behaviors during their past clinic visits.

Any future investigation into adherence to treatment and satisfaction among hypertensive patients and its relation to blood pressure control should seek to fully comprehend the patients’ backgrounds, their understanding of the disease, and how they adhere to their treatment plan. This is an important aspect related to HTN and its ramifications since previous studies have shown that patients' ideas about their diseases and medications are among the most important predictors of adherence to treatment [25-27].

## Conclusions

The results of this study suggest a good level of satisfaction among patients of HTN in Saudi Arabia with respect to the information, simplification, and justification of treatment plans provided by health professionals. This high satisfaction level accounts for the high adherence to treatment plans. The results also indicate that there is a general lack of awareness about the risk factors of HTN.
